# Determinants of tuberculosis treatment outcome under directly observed treatment short courses in Adama City, Ethiopia

**DOI:** 10.1371/journal.pone.0232468

**Published:** 2020-04-29

**Authors:** Tariku Tesema, Dejene Seyoum, Eyasu Ejeta, Reta Tsegaye

**Affiliations:** 1 Tuberculosis and Leprosy Control and Prevention unit, Oromia Health Bureau, Addis Ababa, Ethiopia; 2 Department of Public Health, Institute of Health Sciences, Wollega University, Nekemte, Ethiopia; 3 Department of Medical Laboratory sciences, Institute of Health Sciences, Wollega University, Nekemte, Ethiopia; 4 Department of Nursing, Institute of Health Sciences, Wollega University, Nekemte, Ethiopia; The University of Georgia, UNITED STATES

## Abstract

**Background:**

Tuberculosis (TB) is a leading cause of death among infectious agents, ranking above HIV/AIDS. Though much effort has been done, Ethiopia remained one of those countries which share the greatest burden of TB. Evaluating the TB treatment outcome is one method of TB control measures. Therefore, the aim of the current study was to assess TB treatment outcome and its determinants under directly observed treatment short courses in Adama City, Central Ethiopia.

**Method:**

An institutional based cross sectional study was conducted in all public and private health facilities of Adama city from March 1^st^ 2016 to December 31^st^, 2016. The data were entered and analyzed by using SPSS version 21.0 statistical software. The results were presented using descriptive statistics. Univariate and multivariate logistic regression model was used to evaluate the potential determinants of unsuccessful treatment outcome.

**Results:**

Among 281 patients evaluated, 90(32%) were cured, 137(48.8%) have completed the treatment, 4(1.4%) were treatment failure, 36(12.8%) were lost to follow up, and 14 (5%) died. The overall treatment success rate was 80.8%. Age 15–24 (Adjusted odds ratio (AOR): 4.97; 95% Confidence interval (CI): 1.13–21.90), distance less than 5 kilometers from treatment center (AOR: 3.1; 95% CI: 1.42–6.77), being seronegative for human immunodeficiency virus (HIV) (AOR: 20.38; 95% CI: 7.80–53.24) were associated with successful TB treatment outcome.

**Conclusion:**

The treatment outcome of all forms tuberculosis patients in Adama city was unsatisfactory when referred with the national pooled estimate of 86% and WHO 2030 international target of ≥90%. Thus, enhancing client supervision, treatment monitoring; and working on provision TB treatment services at nearby health facilities should be a priority concern to improve the success rate of treatment outcome. Further studies are also recommended to explore important factors which were not examined by current study.

## Introduction

Tuberculosis (TB) is an old disease which remains the top cause of death as a single infectious disease despite the availability of effective diagnosis tool and treatment. It is mainly caused by the bacillus *Mycobacterium tuberculosis*. The bacterium is mainly transmitted by inhaling droplet aerosols from lungs with active lung disease. It usually affects pulmonary system (pulmonary TB) and occasionally any other anatomic site (extra pulmonary TB) of the body. There were an estimated 10 million new Tb cases, 1.6 million TB deaths, and 558 000 rifampicin resistant TB cases worldwide in 2017 [1–3].

According to the World Health Organization (WHO) report, TB is one of the top ten leading causes of death worldwide. Between 2000 and 2017 alone, an estimated 54 million TB death were prevented by improved disease prevention and treatment, and better service delivery. However, close to 10 million people continued to fall ill with TB in the same year. In 2016 alone, 1.5 million TB deaths were recorded globally of which Africa shares close to three fourth. According to WHO TB report, 20 countries share 85% of all estimated TB cases worldwide and Ethiopia falls under this category which indicates high TB burden in the country [4–6]. Ethiopia is one of the 30 high TB, TB/HIV and multi-drug resistant (MDR-TB) burden countries in the world with more than 126,000 reported cases in 2016.; and despite achieving a treatment success rate of more than 90 percent, 30 percent of cases remain undetected [7]. According to WHO 2014 & 2015 report, the prevalence and incidence of all forms of TB were 211 and 224 per 100,000 of the population, respectively [8].

TB is a curable disease which can be treated with a combination of several drugs for 6 to 12 months. Most commonly TB is treated with isoniazid plus three other drugs consisting rifampicin, pyrazinamide and ethambutol. Between 2000 and 2017, about 54 million lives were saved by TB diagnosis and treatment. The United Nations (UN) Sustainable Development Goals (SDGs) have set the target of ending the TB epidemic by 2030. Promoting directly observed treatment short courses (DOTS) helps to ensure, the right drugs are taken at the right time for the full duration of treatment, and has a paramount importance in increasing TB treatment success rate [9–12]

The WHO currently recommends at least six months of treatment for active disease, and 12 months for latent TB. These long durations of treatment can be difficult for patients to adhere to, especially once they are well and need to return to work. Poor adherence can lead to relapse and even death in individuals, and also has important public health consequences, such as increased transmission and the development of drug resistance. Hence, the WHO recommends that treatment outcome analysis among especially pulmonary TB patients be carried out every year at national and district levels [2, 13].

TB treatment outcomes in Ethiopia were assessed in only limited number of settings, and its success rate varied accordingly based on different factors [14–23]. According to these studies, success rate of TB treatment in Ethiopia ranges from 20% to 92% [14, 19, 21, 24] with varying determinants. In the current study area (Adama city), there is no published data on the TB treatment outcome and associated factors. Therefore, investigating the treatment outcome and identifying its determinants in Adama city could play an important role in improving the TB control strategies and programs of the study area. Thus, this study was aimed to assess the all forms of new tuberculosis treatment outcome and associated factors among TB patients under directly observed treatment short courses in Adama City, Central Ethiopia.

## Materials and methods

### Study area and period

The study was conducted in all public and private health facilities in Adama city, Ethiopia which is situated 99 kilometers South East of country’s capital, Addis Ababa. The area is selected because the city is highly populous and center of trade and main route connecting the country to the outer world. In such cities, where communicable diseases easily spread, evaluating treatment outcomes of TB and its determinants have paramount importance. The data was collected from March 1^st^ 2016 to December 31^st^, 2016

### Study design

An institutional based cross sectional study was conducted at Adama city health facilities.

### Population

#### Source population

All new TB patients attending treatment initiating centers at Adama city from March 1^st^ 2016 to December 30, 2016.

#### Study population

All new TB patients attending treatment initiating centers at Adama city from March 1^st^ 2016 to December 30, 2016.

### Variables

The dependent variable was treatment outcome (successful/unsuccessful) while the independent variables were age, sex, occupation, educational status, religion, distance from health facility, types of TB (smear positive pulmonary TB (PTB), negative PTB, and extra pulmonary TB (EPTB)) and HIV/AIDS serostatus.

### Selection criteria

All new TB patients attending treatment initiating centers at Adama city from March 1^st^ 2016 to December 30, 2016 were included while those patients with incomplete data were excluded.

### Data collection

The data extraction checklists were used for data collection as shown in S1 Table. The data was collected by seventeen diploma nurses after one day training on the data collection technique. The socio demographic data which was not on TB unite registration book like educational status, occupation, and distance from treatment center, income, family size, and religion were collected from the study participants during course of treatment. The principal investigators closely supervised the overall data collection processes.

### Data analysis

The data were entered and analyzed by using SPSS version 21.0 statistical software. Errors related to inconsistency of data such as missing values and outliers were checked and considered during data cleaning. The results were presented using descriptive statistics, and frequency distributions of the variables were computed using tables and figures. To evaluate the potential determinants of unsuccessful treatment outcome, we compared socio-demographic and clinical variables between the successful and unsuccessful treatment outcome groups, using univariate and multivariate logistic regression model. Variables with a P-value less or equal to 0.25 in the univariate analysis were included in the multivariate logistic regression model. Model fitness was checked by using Hosmer-Lemeshow Test of significance and Omnibus test.

### Ethical considerations

The ethical clearance approval was obtained from the Institutional Ethical approval Committee of Wollega University. Letter of support was written to Adama city administration health office and all other TB treatment centers to access patients’ documents, and interview. Written informed consent was taken from study participants and from their parents or guardians for those minors. The information being provided was kept confidential and anonymous. The data were kept in secure place and used only for the intended purpose.

### Operational definitions

**DOTS strategy**: is a new WHO protocol treatment regimen that put the patients under direct observation for the treatment of TB.

**Extra pulmonary TB**: TB which involve other organ except the lungs.

**Pulmonary TB smear positive**: A patient with at least one sputum specimens which are positive for AFB by microscopy.

**Bacteriological confirmed**: A patient with at least one sputum specimens which are positive for AFB by microscopy or positive for AFB in Genxpert or culture test.

**Pulmonary TB- smear negative**: A patient with symptoms suggestive of TB with at least two sputum specimens who were negative for AFB by microscopy and no improvement after tried by broad spectrum antibiotic and with chest radiographic abnormalities consist with active PTB (including interstitial or military abnormal images).

**Drug-resistant TB (DR-TB)** is a general term used to describe a strain of Mycobacterium tuberculosis that is resistant to one or more anti-TB drugs.

**New case (N)**: A patient who has never had treatment for TB or has been on ant-TB treatment for less than four weeks.

**Relapse (R)**: A TB patient who have previously been treated for TB, were declared cured or treatment completed at the end of their most recent course of treatment, and are now diagnosed with a recurrent episode of TB.

**Treatment after Failure (F)**: Treatment after failure patients are those who have previously been treated for TB and whose sputum was positive at the end of five & six months.

**Treatment after Loss to follow-up (L)**: patients who have previously been treated for TB for more than one month and stops treatment for more than 8weeks and were declared lost to follow-up at the end of their most recent course of treatment (Previously known as ‘treatment after default’).

**Treatment outcome**: it is the result obtained after the initiation of the treatment of TB (cured treatment complete, treatment failure, died, lost to follow up, and moved to MDT–TB Register).

**Treatment completed**: A TB patient who completed treatment without evidence of failure but with no record to show that sputum smear or culture results in the last month of treatment and on at least one previous occasion were negative, either because tests were not done or because results are unavailable.

**Cured**: A patient whose sputum smear or culture was positive at the beginning of the treatment but who was smear or culture-negative in the last month of treatment and on at least one previous occasion.

**Treatment success**: Defined as cure or a patient whose sputum smear or culture was positive at the beginning of the treatment but who was smear- or culture-negative in the last month of treatment and on at least one previous occasion plus treatment completion without confirmation by smear microscopy cured or complete treatment.

**Treatment Failure (F)**: A patient whose sputum smear or culture is positive at 5 months or later during treatment or patients found to harbor a multidrug-resistant (MDR) strain at any point of time during the treatment, whether they are smear-negative or -positive.

**Died**: A TB patient who dies for any reason before starting or during the course of Treatment.

**Lost to follow up**: a TB patient who was treated at least for one month and whose treatment was interrupted for two consecutive months or more and declared lost follow up in their recent treatment outcome.

**Successful treatment outcome**: the sum of cured and treatment completed.

**Unsuccessful treatment outcome**: the sum of lost to follow up, died, and treatment failure.

## Results

### Socio-demographic characteristics

A total of 281 TB patients were registered at Adama city in 6 public, 10 private and 1 non-governmental organization health facilities from March 1^st^ 2016 to December 30, 2016. Among these, more than half 157 (55.9%) were males and 171 (60.9%) were urban residents. One hundred twelve (39.9%) of the patients were in the age group 25–34 and the mean age of the respondents was 32.4 years. Nearly half (48.8%) were daily laborers and 59.4% of the respondents were earning a monthly income of less than 600 Ethiopian Birr. Nearly two-third (62.5%) of the respondents was at a distance of more than 5 kilometers from the health facilities. Nearly half (47.7%) of the respondents attended primary education and 61.2% of them were Orthodox religion followers (Table 1).

**Table 1:** Socio demographic characteristics of TB patients who were enrolled for treatment at Adama city health facilities, Central Ethiopia, from March 1st 2016 to December 30, 2016.

Socio demographic variables	Characteristics	Frequency	Percent
**Age**	<5yrs	4	1.4
	5-14yrs	13	4.6
	15–24	102	36.3
	25–34	112	39.9
	≥35years	50	17.8
**Sex**	Male	157	55.9
	Female	124	44.1
**Residence**	Urban	171	60.9
	Rural	110	39.1
**Occupation**	Employed	31	19
	Merchant	68	24.2
	Farmer	34	12.1
	Daily laborer	137	48.8
	Others*	11	1.1
**Income per month (Ethiopian Birr)**	Less than 600	167	59.4
	600–1000	21	7.5
	1001–1500	29	10.3
	1501–2000	42	14.9
	2001–2500	12	4.3
	2501–3000	1	0.4
	3001–3500	5	1.8
	3501 and above	4	1.4
**Distance from treatment center**	≤ 5km	121	43
	5km above	160	57
**Educational status**	No formal education	67	23.8
	Primary school	134	47.7
	Secondary & Above	80	28.5
**Family size**	≤ 5	79	32.2
	Above 5	202	71.9
**Contact person**	HEW	9	3.2
	Health care worker	5	1.8
	Family member	259	92.2
	Self	8	2.8

Others*- no paid job, retiree.

### Clinical characteristics

**Fig 1:**
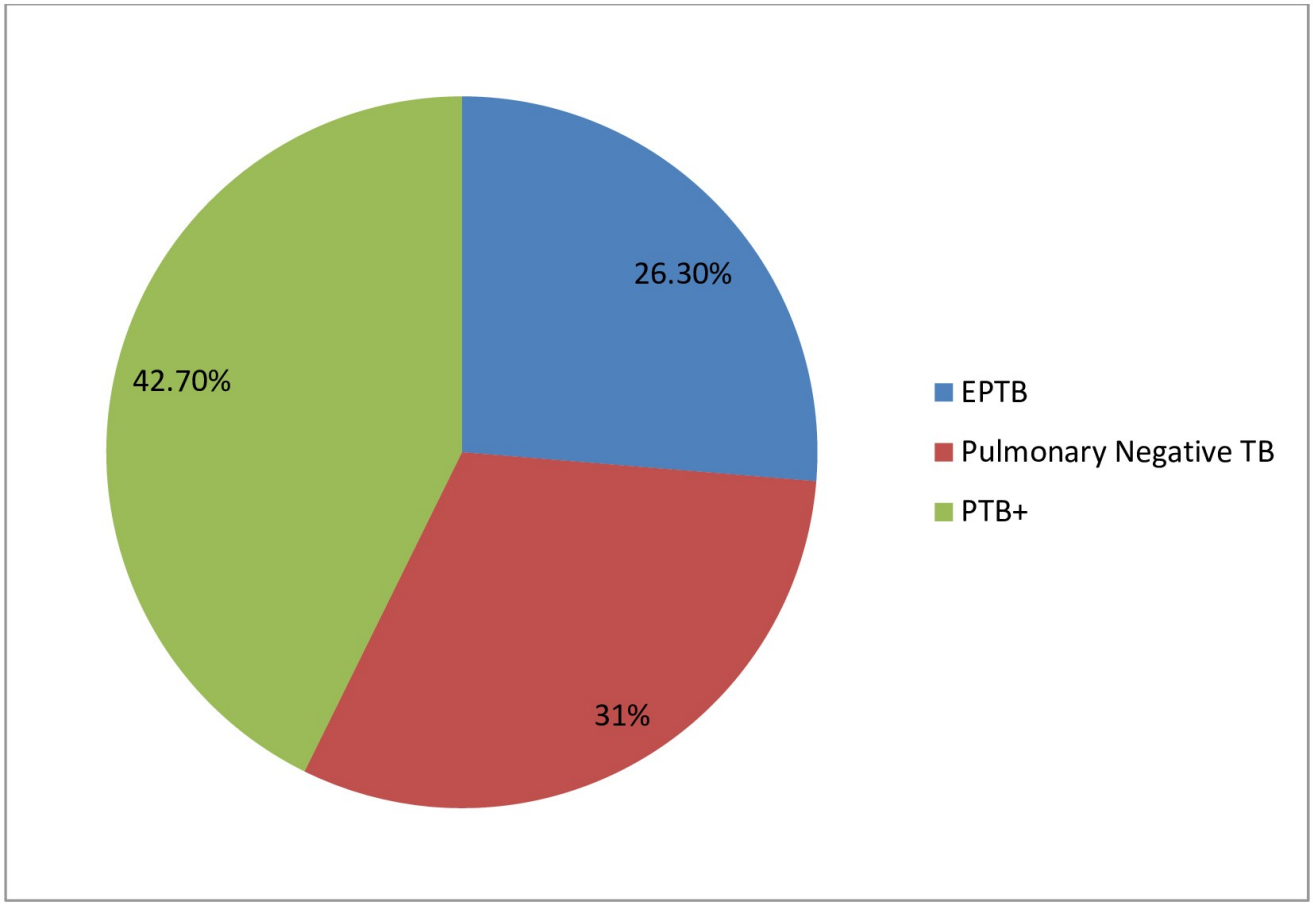
Types of TB cases among TB patients treated at Adama city health facilities, Central Ethiopia, March 1st 2016 to December 31, 2016. (One hundred twenty (42.7%) of the study participants were smear positive pulmonary TB (PTB+), 87 (31%) were smear negative pulmonary TB and 74(26.3%) were extra pulmonary TB).

Among the study participants, 120 (42.7%) were bacteriologically confirmed smear positive, 87(31%) were clinically diagnosed Pulmonary Negative (P/Neg) TB and the rest 74 (26.3%) were extra pulmonary TB (Fig 1).

From the total patients registered during the study period, 244 of them were non-reactive for HIV while 35(12.4%) were TB/HIV co-infected and the remaining two (0.7%) were unknown status. Sputum smear tests were done at second and fifth months. The result of the smear conversion at second month showed that 106 (88.3%) of them were negative, 10 (8.3%) Positive and 4(3.3%) of them were missing. Also at 5th month, 112(93.3%) of them were negative, 4(3.3%) were positive, and 4(3.3%) were missing.

### Treatment outcome

**Fig 2:**
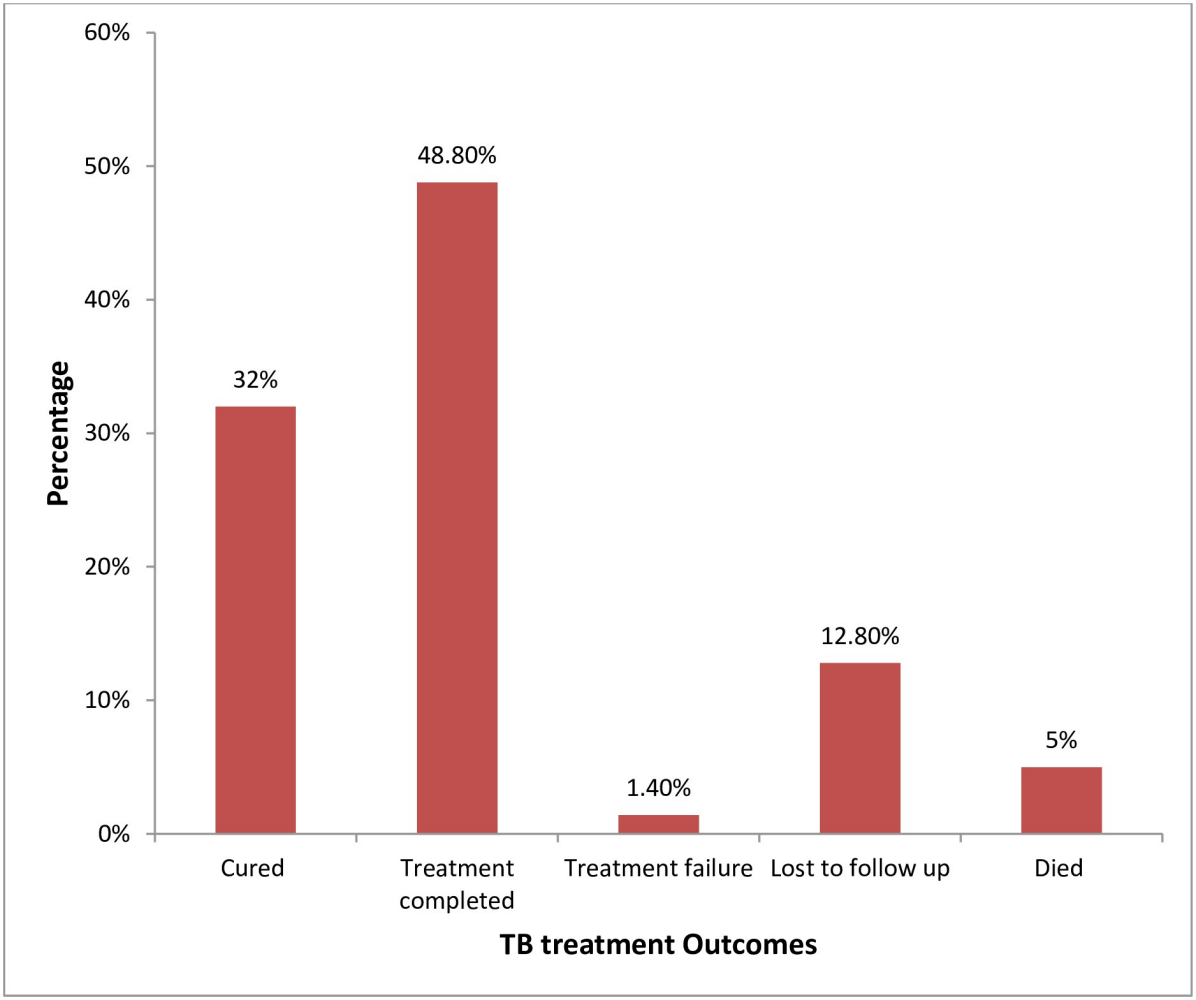
Treatment outcome of TB patients treated at Adama city health facilities, Central Ethiopia, March 1st 2016 to December 31, 2016. (Among study participants, nearly half (137(48.8%)) of them completed the treatment, 90(32%) were cured, 36 (12.8%) lost to follow up, 4(1.4%) were treatment failure, and 14(5%) died).

After six months of treatment, outcomes of TB patients were assessed. Accordingly, 90(32%) were cured, 137(48.8%) have completed the treatment, 4(1.4%) were treatment failure, 36(12.8%) were lost to follow up, and 14 (5%) died (Fig 2).

The overall treatment success rate was (80.8%). Among bacteriologically confirmed TB patients, 90(75%) were Cured, 13(10.8%) treatment completed while 4(3.3%) were treatment Failure, 11(9.2%) were lost to follow up and 2 (1.6%) died. From clinically diagnosed Pulmonary Negative, 66 (75.8%) have completed their treatment, 15(17.24%) were lost to follow up, 6(6.9%) died, and among extra pulmonary TB, 57(78.4%) have completed the treatment, 11 (14.9%) were lost to follow up and 6 (5.4%) died.

### Factors associated with TB treatment outcome

The treatment outcome was varied with age, distance from health facility, and HIV serostatus. The odds of successful treatment outcome were 4.97 times higher among patients 15–24 years of age compared to patients younger than 14 years of age (AOR: 4.97; 95% CI: 1.13–21.90). Patients were 3.1 times more likely to have successful treatment outcome if they had to walk for less than five kilometers to the treatment center than those who are far from the treatment center of more than five kilometers (AOR: 3.1; 95% CI: 1.42–6.77). HIV negative patients were about 20 times more likely to have favorable treatment outcome than HIV patients co-infected with TB (AOR: 20.38; 95% CI: 7.80–53.24) (Table 2). There was no significant difference on the outcome of TB treatment based on sex, residence; occupational status and educational status.

**Table 2:** Factors associated with treatment outcome among TB patients at Adama city health facilities, central Ethiopia from March 1^st^ to December 30, 2016.

Characteristics	TB treatment outcome		COR [95% CI]	P-value	AOR [95% CI]	P-value
	successful	unsuccessful				
**Age**						
< 15	12 (70.6)	5 (29.4)	1.00		1.00	
15–24	90 (88.2)	12 (11.8)	3.1 (0.94–10.42)	0.06	4.97 (1.13–21.90)*	**0.034**
25–34	87(77.7)	25(22.3)	1.45(0.47–4.51)	0.52	1.92(0.48–7.66)	0.358
≥ 35	38(76.0)	12(24.0)	1.32(0.39–4.51)	0.66	1.1(0.26–4.75)	0.904
**Residence**						
Urban	145(84.8)	26(15.2)	1.90(1.05,3.47)	0.035	1.46(0.68–3.13)	0.338
Rural	82(74.5)	28(25.5)	1.00		1.00	
**Distance from health facility**						
>5 KMs	84(69.4)	37(30.6)	1.00		1.00	
≤5 KMs	143(89.4)	17(10.6)	3.71(1.97,6.99)	<0.001	3.1(1.42–6.77)*	**0.004**
**Occupation**						
Employee	29(10.3)	2(0.7)	1.00		1.00	
Unemployed	198(70.5)	52(18.5)	0.263(.061–1.137)	0.074	0.51(0.1–2.58)	0.417
**Category of TB**						
Bacteriological confirmed p/pos.	104(37.01)	16(5.7)	1.00		1.00	
Clinically confirmed P/Neg	65(23.13)	22(7.8)	0.46(0.22, 0.93)	0.031	0.6(0.25, 1.44)	0.252
**EPTB**	58(20.64)	16(5.7)	0.56(0.26, 1.20)	0.134	0.43(0.17, 1.10)	0.080
**HIV serostatus**						
Reactive	11(3.9)	24(8.5)	1.00		1.00	
Non-reactive	215(76.9)	29(10.7)	16.2(7.2,36.4)	<0.001	20.38 (7.80–53.24)*	**0.000**
**Unknown status**	1(0.36)	1(0.36)	2.2(0.13, 38.18)	0.593	2.9(0.09, 94.30)	0.550

*shows statistically significant association.

## Discussion

Assessment of anti-tuberculosis treatment outcome as well as analysis of factors responsible for poor treatment outcome was one of the major indicators for the evaluation of the performance of a national TB program. In this study, information on the treatment outcome of all types of TB patients across the study period was assessed in Adama city health facilities, Central Ethiopia. The overall treatment success rate for all cases of tuberculosis in our study was 80.8%. This supports previous studies in Ethiopia with success rates of 80.2% in Jimma, 81.5% in Sodo town, 79.6% in Addis Ababa and 82.9% in Assela [15, 18, 22, 25]. This result shows a slight improvement when compared with retrospective cross-sectional survey done on TB treatment outcomes in Pakistan that showed 68% treatment success rate [24], and Jinka general hospital with success rate of 74% [16] but lower than studies conducted in East Wollega (91.9%) [14], Gambella regional state (88.1%) [17], twenty two districts of Ethiopia (90.9%) [19] and Debre Tabor (87.1%) [20]. This difference might be because of geographical variation between the study settings, difference in sample size and variation of study period. The success rate in the current study was also lower than the pooled estimate of TB treatment success rate in Ethiopia (86%) and WHO 2030 international target of ≥90% [4, 26].

According to this study, age of the patient is one of the significant predictor for unsuccessful treatment outcome. The odds of successful treatment outcome were 4.8 times higher among patients 15–24 years of age compared to patients younger than 14 years of age (AOR: 4.81; 95% CI: 1.09–21.06). This may be due to younger population has capability to reach to health facility, interest to adhere to their medication follow up, and has better immunity than older people and children. A retrospective study conducted in South Africa and Assela teaching hospital similarly revealed that treatment failure was less likely among youths than older adults [22, 27]. A five year retrospective study conducted in Addis Ababa also shown that patients older than 25 years had a significantly low treatment success rate compared to the other age group [28]. The possible variation of treatment outcome based on age could be due to older individuals often have associated diseases, general physiological deterioration along with age, less able to reach health care facilities and are also poorer than the younger individuals.

The result of the current study also indicated that HIV/AIDs had statistically significant impact on TB treatment outcome such that co-infected individuals had unsuccessful treatment outcome compared to HIV sero-negative patients (p<0.001). This result is similar with previously conducted studies in different parts of Ethiopia. A study conducted in Eastern part of Ethiopia revealed that being HIV seropositive increases the likely hood of treatment failure [23]. Similarly, studies conducted in Nairobi, Gambella region, Dire Dawa, Assela, and Harar town shown that TB/HIV co-infection had significant influence on successful TB treatment outcome [6, 17, 22, 29, 30]. This could be due to the fact that mortality as a result of co-infection is higher than TB infection alone and taking many antiretroviral and anti-TB tablets daily can be difficult and challenging to a patient that might result in treatment failure.

The patient near to the treatment center (less than 5km) had good treatment successes when compared to those greater than 5km from the facility. This result is consistence with a systematic review and meta-analysis on prevalence and determinants of anti-tuberculosis treatment non-adherence in Ethiopia which revealed, feeling distance to health institution is long were found to be determinants of failure to tuberculosis treatment [31]. A study conducted in Kenya similarly shown that traveling away from treatment site was a significant factor for the TB treatment default [29]. This implies that coverage of health care institutions including rural settings where the community can access health care services nearby should get due attention to increase success rate of TB treatment outcome.

## Limitations

Even though some data which were not available from patient registration were collected from the patient itself, the study has still limitation. The main limitation was the study only included the new TB cases and re-treatment cases were not included. Factors like substance abuse and presence of underlying chronic disease were also not investigated in this study. Thus, the findings of the current study should be interpreted by taking the above limitation in to consideration.

## Conclusions

The treatment outcome of all forms tuberculosis patients in Adama city was unsatisfactory when referred with the national pooled estimate of 86% and WHO 2030 international target of ≥90%. This could be due to factors that were significantly associated with TB treatment outcome like age, co-infection with HIV, and distance from the treatment center. Thus, enhancing client supervision, treatment monitoring; and working on provision TB treatment services at nearby health facilities should be a priority concern to improve the success rate of treatment outcome. Further studies are also recommended to explore important factors which were not examined by current study.

## Supporting information

S1 Table Data extraction checklist for determinants of tuberculosis treatment outcome under directly observed treatment short courses in Adama City, Central Ethiopia. (PDF)
