# Supplementary material for: Determinants of tuberculosis treatment outcome under directly observed treatment short courses in Adama City, Ethiopia
Source: PLoS One. 2020 Apr 29;15(4):e0232468. doi: 10.1371/journal.pone.0232468 (PMC7190142; doi:10.1371/journal.pone.0232468)
Supplement: S1 Table — (PDF) [file pone.0232468.s001.pdf]

Data Collection Instrument /Checklist and questionnaire

Date of data collection [___/___/_____] (dd/mm/yyyy)

Name of the data collector _________________________________ Signature _____________

Time (Started/ Ended) [___/_____; ____/______] (hr. /min)

For supervisor only:<br>Any incomplete or inconsistent data available: Yes/No<br>If yes, type and action taken: ____<br>____<br>*(Please use additional blank paper if the space is not enough)*<br>Supervisor Name __________________________________<br>Signature ________________ Date [___/____/_______] (dd/mm/yyyy)

Check list

**Part 1: Socio Demographic Information**		
**S. No**	**Questions**	**Response**
1.1	Name of the treatment initiating center	.... hospital/health center
1.2	Type of treatment initiating center	Ambulatory model of care------------------1<br>Hospitalized/inpatient model of care------2
1.3	Patient - TB registration number	-------------------------------------
1.4	Patient medical record number (MRN)	-------------------------------------
1.5	Patient unique number(Region/type of facility/facility code/ number)	-------/--------------/-------/DR----------------
1.6	Age	.... in complete Years
1.7	Sex	Male--1<br>Female---------------------------------------2
1.8	Address	Urban---1<br>Rural--2

1.9	Distance from treatment center	
1.10	Educational Status	
	Religion	Orthodox----------------1<br>Protestant----------------2<br>Catholic------------------3<br>Musilim-------------------4<br>Others----------------------5
1.11	Family size	Two-----------1<br>Three----------2<br>Four and above-----3
1.12	Is there TB case history in your family	On Treatment in this time-----1<br>Previously treated --------------2
1.13	Occupation/employment	Governmental employed-----1
		Private employed/NGO--------2
		Merchant-------------------------3
		Farmer----------------------------4
		Daily laborer and other-------------5
1.14	Income/per month	In Ethiopia birr--------------
1.15	Do you know TB is curable	Yes---------------1<br>No----------------2<br>I am not sure -----3
1.16	Is TB Drugs are safe?	Yes---------------1<br>No----------------2<br>I am not sure -----3
**Part 2: clinical Conditions at initiation, during treatment and at the end of intensive of treatment**		
2.1	Has the patient contact person/ treatment supporter	Yes--1<br>No --2

2.2	If yes to question no 2.1, who was/is contact person/treatment supporter?	HEW--1<br>Health care worker---------------------------2<br><br>Family member-------------------------------3<br>Self administer--------------------------------4<br>Health development army member---------5<br>Other(specifiy)-------------------------98<br>Missing …………………………….99
2.3	Category of –TB	Smear positive pulmonary TB-------1<br>Smear negative pulmonary -TB------2<br>Extra pulmonary -TB-----------------3
2.4	Has the patient had previous history of first line TB treatment	Yes--1<br>No---2<br>Missing------------------------------------99
2.5	If yes to 2.7, what were/was the frequency of treatment?	One times-------------------------------------1<br>Two times-------------------------------------2<br>Three times-----------------------------------3<br>Missing ---------------------------------------99
2.6	What was the patient category before treatment initiation	New(N)---1<br>Relapse(R)-------------------------------------2<br>Treatment after failure of New TB regimen---3<br>Treatment after failure of Retreatment regimen---4<br>Treatment after being lost to followup-----5<br>Transfer in(T)--------------------------------6<br>Others(O)-------------------------------------7<br>Missing--------------------------------------99

2.7	TB diagnosed date	…../…./….. (dd/mm/yyyy)
2.8	TB treatment started date	…../…./….. (dd/mm/yyyy)
2.9	Category of treatment	**New:** 2RHZE/4RH …….1<br><br>**Previously treated:** Treatment after failure:- 2RHZES/1RHZE/5RHE*) ……2*
2.10	TB treatment regimen	………………………..(Write the name of drugs)
2.10.1	Intensive phase	Yes---1<br>No--2<br>Missing ---------------------------------------99
2.10.2	Continuation phase	Yes---1<br>No--2<br>Missing ---------------------------------------99
2.11	Was/is the -TB treatment regimen changed?	Yes---1<br>No--2<br>Missing--99
2.12	If yes to number 2.11,name of changed -TB treatment regimen(drugs)	----,--------,---------,---------,---------,----<br>drugs(name of drug in abbreviation)
2.13	If yes, what was/is the reason?	Due to side effect----------------------------1<br>Due to other disease-----------------------2<br>Due to sotck out----------------------------98<br>Missing …………………………….99

2.14	Weight(kg)of the patient at respective months of treatment	-------kg at initiation of treatment(zero months)<br>.......... kg at 1st month of treatment<br>......... kg at 2th month of treatment<br>.......... kg at 3th month of treatment<br>.......... kg at 5th month of treatment<br>.......... kg at 6th month of treatment<br>--------kg at 8th month of treatment etc.
2.15	Was the patient tested for HIV/AIDS?	Yes……………………………………1<br>No---2<br>Missing …………………………………99
2.16	If yes to question no 2.19,what was/is the HIV/AIDS status of the patient	Reactive…………………………………1<br>Non-reactive……………………………..2<br>Indeterminate……………………………3<br>Unknown………………………………..4
2.17	If yes to number 2.20 above, what is/was the CD4 count (write the actual number or % for children) at :	Baseline=---------1<br>Six months=--------2<br>12 months=------------3<br>Missing ………………99
2.18	If reactive for question number 2.21, is/was the patient put on Cotrimozale preventive therapy (CPT)	Yes ------------1[write CPT started date (DD/MM/YY)____/_____/_______]<br>No……………………………………..2<br>Missing ……………………………….99
2.19	If reactive for question number 2.22, is/was the patient linked to the chronic HIV/AIDS care (pre-ART)?	Yes ------------1[write the date on which linked to chronic care (DD/MM/YY)____/_____/_______]<br>No………………………………..……...2<br>Missing ………………………………..99

2.20	If reactive for question number 2.23, is/was the patient initiated on ART?	Yes -------------1[write the date on which initiated on ART (DD/MM/YY)____/_____/_______]<br>No…………………………………………2<br>Missing …………………………………99
2.21	Smear conversion follow up result (only for sputum smear positives PTB):<br>Positive--------------------------------1<br>Negative-------------------------------2<br>Not done------------------------------3<br>Unknown------------------------------4<br>Missing---------------------------------5	.........at 2th month of treatment<br>.........at 3th month of treatment<br>......... at 5th month of treatment<br>..........at 6th month of treatment<br><br>------- at 8th month of treatment etc.
2.22	If suspected MDR X/Pert or Culture result at result:<br>(positive------------------------------1<br>Negative------------------------------2<br>Not done------------------------------3<br>Unknown-----------------------------4<br>missing--------------------------------5	------at initiation of treatment(zero months)<br>.........at 2th month of treatment<br>.........at 3th month of treatment<br>..........at 5th month of treatment<br>..........at 6th month of treatment<br><br>-------- at 8th month of treatment etc.
2.26	What is patient’s treatment outcome at the end of 6th month (new cases) of treatment	cured -----------------1<br>treatment complete ------------2<br>treatment failure-----------------------------3<br>Lost to follow up-----------------------------4<br>Transfer out-----------------------------------5<br>Died ---6<br>Transfer to MDR RX initiating center …7<br>(write date of death:DD/MM/YY=-----/-----/----)7

2.27	Treatment outcome of the at 8th months (retreatment TB cases)	Cured--1<br>Treatment completed------------------------2<br>Failed--3<br>Lost to follow up-----------------------------4<br>Died--5<br>Transfer out-----------------------------------6<br>Transfer to MDR RX initiating center …7<br>Unkown--8<br>Other(specify):-------------------------------98<br>Missing---------------------------------------99
